# Viral Genotypes and Associated Risk Factors of Hepatocellular Carcinoma in India

**DOI:** 10.7497/j.issn.2095-3941.2012.03.004

**Published:** 2012-09

**Authors:** Manash Pratim Sarma, Mohammad Asim, Subhash Medhi, Thayumanavan Bharathi, Richa Diwan, Premashis Kar

**Affiliations:** 1 PCR Hepatitis Laboratory, Department of Medicine, Maulana Azad Medical College, University of Delhi, New Delhi 110002, India; 2 Department of Gastroenterology, Rajaji Government Hospital, Madurai 625020, India

**Keywords:** hepatocellular carcinoma, hepatitis B virus, hepatitis C virus, risk factors

## Abstract

**Objective:**

This study aims to investigate the etiological relationship among hepatitis B virus (HBV), hepatitis C virus (HCV), and alcohol as risk factors in a cohort of hepatocellular carcinoma (HCC) patients from India. The clinical and biochemical profiles and tumor characteristics in the HCC cases were also evaluated.

**Methods:**

A total of 357 consecutive cases of HCC fulfilling the diagnostic criteria from the Barcelona–2000 EASL conference were included in the study. The blood samples were evaluated for serological evidence of HBV and HCV infection, viral load, and genotypes using serological tests, reverse transcription-polymerase chain reaction, and restriction fragment length polymorphism.

**Results:**

The male/female ratio for the HCC cases was 5.87:1. Majority of the HCC patients (33.9%) were 50 to 59 years of age, with a mean age of 4±13.23 years. More than half the cases (60.8%) had underlying cirrhosis at presentation. Among the HCC patients, 68.9% were HBV related, 21.3% were HCV related, 18.8% were alcoholic, and 18.2% were of cryptogenic origin. The presence of any marker positive for HBV increased the risk for developing HCC by almost 27 times [OR: 27.33; (12.87–60.0)]. An increased risk of 10.6 times was observed for HCC development for cases positive for any HCV marker [OR: 10.55; (3.13–42.73)]. Heavy alcohol consumption along with HCV RNA positivity in cirrhotic patients was found to be a risk for developing HCC by 3 folds [OR: 3.17; (0.37–70.71)].

**Conclusions:**

Patients of chronic HBV infection followed by chronic HCV infection were at higher risk of developing HCC in India. Chronic alcohol consumption was found to be a risk factor in cirrhotic cases only when it was associated with HCV RNA positivity. Most of the patients had a large tumor size (>5 cm) with multiple liver nodules, indicating an advanced stage of the disease thus making curative therapies difficult.

## Introduction

Hepatocellular carcinoma (HCC) is one of the major causes of morbidity worldwide^[^^1^^]^. It represents the third leading cause of cancer death in males and the fourth in females, with more than 600,000 deaths per year^[^^2^^]^. The geographical distribution of HCC varies throughout the world, with an incidence ranging from 2.1 per 100 000 in Central America to 35.5 per 100 000 in Eastern Asia^[^^3^^]^. Globally, three epidemiological zones have been defined according to the age-adjusted HCC incidence per 100 000 inhabitants per year: low (<5%), intermediate (5% to 15%), and high (>15%)^[^^4^^]^. Levrero et al.^[^^5^^]^ reported that a geographical correlation exists between the incidence of HCC and the prevalence of chronic hepatitis B and C viruses, suggesting that these two viral infections are the most important risk factors associated with HCC. In countries where hepatitis C virus (HCV) infection is endemic (e.g., Japan and Egypt), a high prevalence of HCV infection is reported among people with HCC. Meanwhile, hepatitis B virus (HBV) infection is the major risk factor associated with the development of HCC in regions with large populations (e.g., China and Southern Asia) because of its high endemicity^[^^1^^]^. In addition to the viral infections largely implicated in HCC development, other factors associated with HCC are well documented. These factors include toxins (e.g., alcohol consumption) and drugs (e.g., aflatoxin and anabolic steroid use), cigarette smoking, metabolic liver diseases (e.g., hereditary hemochromatosis, and alpha1-antitrypsin deficiency), and steatosis^[^^6^^,^^7^^]^. Some of these factors have a direct carcinogenic role, whereas others interact by promoting fibrosis and cirrhosis^[^^8^^]^. Recent studies found a significant association between non-insulin-dependent diabetes (NIDD or type II diabetes) and HCC, suggesting that diabetes is a potential risk factor for HCC development^[^^6^^,^^9^^]^.

India falls in the low HCC incidence zone^[^^10^^,^^11^^]^. In most patients, the development of HCC is closely associated with liver cirrhosis. The three main causes of HCC are HBV, HCV, and alcohol, among which HBV seems to play a direct role in liver cell transformation^[^^12^^]^. HCV was found in some HCC patients without cirrhosis^[^^13^^]^; however, its carcinogenic role in the absence of cirrhosis is controversial. Although alcohol is proposed to cause HCC because it causes cirrhosis, its association with HCC in the absence of cirrhosis remains unknown^[^^14^^]^.

Little is known regarding the etiology and clinical, biochemical, and radiological profiles of HCC cases and their survival data from India. This study aims to investigate the risk factors of HCC and the clinical, biochemical, and radiological profiles of 357 HCC cases from India.

## Patients and Methods

A total of 357 HCC cases were included in the study during the period between 2003 and 2010. Cases from the medicine OPD of Lok Nayak Hospital (New Delhi) and from the gastroenterology OPD of Rajaji Hospital (Madurai, India) were included. The diagnoses of HCC and chronic hepatitis were based on the criteria from the Barcelona–2000 EASL conference and from the recommendation of AASLD 2009 updated guidelines, respectively^[^^15^^]^. Cirrhosis was diagnosed based on morphological and clinical criteria, as well as ultrasound or Computed Tomography (CT), according to standard definitions^[^^16^^]^. An equal number (357) of age and sex-matched cases of chronic hepatitis and cirrhosis of liver without HCC served as the control group. A second control group including 120 healthy cases without any history of liver diseases was used to compare the risk factors associated with HCC.

Written informed consent was obtained from all the subjects. The study protocol conformed to the ethical guidelines of the 1975 declaration of Helsinki and was approved by the ethics committees of both centers. Blood samples for serological analysis were collected, and the participants were interviewed using a standard questionnaire that included questions about clinical symptoms and medical history. All clinical, biochemical, serological, radiological, and cytohistological details were noted from the case records.

The cases were evaluated based on history, physical examination, and liver function profile. Serological tests for the detection of hepatitis B and C and estimation of serum α-fetoprotein (AFP) levels were performed using commercially available third-generation enzyme-linked immunosorbent assay. Specialized investigation included endoscopy, abdominal ultrasound, triphasic CT, and magnetic resonance imaging (MRI). Genotyping and quantification of HBV and HCV were conducted using polymerase chain reaction (PCR)-restriction fragment length polymorphism (RFLP) and real time PCR, respectively.

### Serological examination

HBsAg and IgG anti-HBc were detected with enzyme immunoassay (EIA; Abbott Laboratories, Abbott Park, IL). Samples positive for HBsAg or anti-HBc antibodies or both were considered positive for HBV. Anti-HCV was detected with HCV EIA version 3.0 (Abbott Laboratories). Liver function tests of all the samples were estimated using an auto-analyzer (Hitachi, Tokyo, Japan). Serum AFP levels were determined using an Immulite-100 automated immunoassay system (Diagnostic Products, Los Angeles, CA, USA).

### HBV detection and genotyping

HBV DNA was extracted using a QIAamp DNA Mini Kit (Qiagen Inc, Chatsworth, CA) and detected by PCR using primers specific for the S and pre-C/C regions of the HBV genome. Serum HBV-DNA levels were quantified using a branched-DNA assay (Quantiplex HBVDNA; Chiron Corp., Emeryville, CA, USA).

### HCV detection and genotyping

HCV-RNA was extracted from serum samples by TRIzol LS reagent (GIBCO BRL, Life Technologies, Maryland, MD, USA). HCV genotyping was performed through the RFLP method described by Chinchai et al.^[^^17^^]^ using the enzymes AccI, MboI, and BstN1. The results of the mixed-genotype infection by RFLP typing were evaluated followed by direct sequencing.

### Statistical analysis

The odds ratio (OR) with 95% confidence interval for the risk factors of HCC were calculated by logistic regression using the SAS statistical package. The rates and ratios were compared using the χ^2^ test. SPSS 11.5 statistical software was used for calculations; *P*<0.05 indicate statistical significances.

## Results

### Baseline characteristics of HCC patients

In this study, the male/female ratios in the HCC group and in the control group were 5.87:1 and 2.31:1, respectively. Majority of the HCC patients (33.9%) and control subjects (38.4%) were 50 to 59 years and 40 to 49 years of age, respectively (**Table 1**). The demographic details of the healthy control group were similar to those of the other control group with slight variations. The mean age of the HCC patients was (54±13.23) years. Approximately 90.5% of the HCC cases were symptomatic, whereas the remaining 9.5% were asymptomatic. Approximately 10% of the HCC cases were known cases of cirrhosis, whereas another 60.8% had underlying cirrhosis at presentation. Among the 357 cases, 108 (30.3%) were smokers, 249 (69.75%) were non-smokers. Alcohol consumption was documented in 59.1% of the cases, whereas the rest were non-alcoholic (**Table 2**). Conclusive evidence of liver cirrhosis was reached in 280 cases (78.4%), of which 176 (62.8%) had HCC and 104 (27.2%) had no HCC.

**Table 1:** Demographic characterization of HCC and control patients.

Characteristics	Healthy controls (*n*=120*)*	HCC (*n*=357*)*	Controls^†^ (*n*=357*)*
Gender			
Male	71 (59.17)	305 (85.43)	249 (69.75)
Female	39 (40.87)	52 (14.57)	108 (31.25)
Age (years)			
0-9	0 (0)	0 (0)	0 (0)
10-19	3 (2.50)	4 (1.12)	6 (0.16)
20-29	7 (5.83)	21 (5.88)	20 (5.60)
30-39	18 (15.0)	41 (11.48)	38 (10.64)
40-49	31 (25.83)	66 (18.49)	137 (38.37)
50-59	42 (35.0)	121 (33.89)	72 (20.17)
60-69	8 (6.67)	69 (19.32)	60 (16.80)
70-79	1 (0.83)	32 (8.96)	24 (6.72)
80-89	0 (0)	3 (0.84)	0 (0)

†: chronic hepatitis cases without HCC.

**Table 2:** Baseline characteristics of HCC patients in India.

Characteristics	
Mean age (years)	54±13.23
Sex ratio (M:F)	5.87:1
Symptomatic HCC	323 (90.48)
Asymptomatic HCC	34 (9.52)
Portal Hypertension	269 (75.35)
Known cirrhotic at diagnosis	36 (10.08)
Underlying cirrhotic at diagnosis	217 (60.78)
Smoking status	
Smokers (20/day for 10 years)	108 (30.25)
Non smokers	249 (69.75)
Alcohol intake	
Alcoholics	211 (59.10)
Non alcoholics	146 (40.90)

### Clinical profiles of HCC cases and controls

Symptoms such as jaundice, nausea, hepatic encephalopathy, and melena were present in almost equal proportion in HCC patients and controls. Other symptoms such as general weakness (59.4% in HCC *vs.* 19.9% in controls), weight loss (71.7% in HCC against 45.4% in controls), abdominal discomfort (82.4% in HCC *vs.* 53.5% in controls), and anorexia (64.4% in HCC *vs.* 40.9% in controls) were more frequently noticed in the HCC cases than in the controls. Among the 357 cases of HCC, 133 (37.3%) had ascites, 187 (52.4%) had hepatomegaly, 162 (45.4%) had pallor, 101 (28.3%) had pedal edema, 142 (39.8%) had spleenomegaly, and 114 (31.9%) had icterus. In the controls, the above-mentioned signs were documented in more or less similar proportion with that of the cases (**Table 3**).

**Table 3:** Clinical profile of HCC and control cases.

	HCC, *n*=357 (%)		Controls^†^, *n*=357 (%)		*P*
Signs					
Ascites	133 (37.25)		74 (20.73)		0.000000546
Hepatomegaly	187 (52.38)		177 (49.58)		0.2277
Pallor	162 (45.38)		115 (32.21)		0.0001559
Pedal Edema	101 (28.29)		67 (18.77)		0.001371
Icterus	114 (31.93)		92 (25.77)		0.03500
Spleenomegaly	142 (39.78)		97 (27.17)		0.0001820
Symptoms					
Jaundice	101 (28.29)		85 (23.81)		0.08698
Weakness	212 (59.38)		71 (19.88)		<0.0000001
Weight loss	256 (71.70)		162 (45.38)		<0.0000001
Abdomen discomfort	294 (82.35)		191 (53.50)		<0.0000001
Anorexia	230 (64.4)		146 (40.90)		<0.0000001
Nausea	84 (23.53)		78 (21.85)		0.2967
Hepatic Encephalopathy	16 (4.48)		7 (1.96)		0.02994
Malena	40 (11.20)		28 (7.80)		0.06431

†, chronic hepatitis cases without HCC

### Viral etiology and the risk factors of HCC

Of the HCC patients, 68.9% were HBV related and 21.3% were positive for HCV markers. Coinfection of HBV and HCV was observed in 5.3% of the HCC cases. A total of 18.8% of the cases were alcoholic, whereas 18.2% of the cases were of cryptogenic origin (**Table 4**). In both groups, HCV genotyping showed that genotype 3 was the major genotype, followed by genotypes 1 and 4 (**Table 5**). HBV genotype D was the most prevalent, followed by A, whereas a mixed genotype of A + D was documented in 16.6% of the cases. Majority of the HCC cases had a high viral load of HBV (**Table 6**).

**Table 4:** Prevalence of viral marker and heavy alcohol use among patients with hepatocellular carcinoma.

Causative factors					HCC cases (%)	
HBV markers						
Overall		246 (68.91)			
HBsAg+		201 (56.30)			
Only Anti HBe+		17 (4.76)			
Only IgG Anti HBc+		28 (7.84)			
HCV markers						
Overall		76 (21.29)			
Anti HCV+ve & HCV RNA+ve		66 (18.49)			
Anti HCV+ve & HCV RNA-ve		10 (2.80)			
HBV & HCV	19 (5.32)					
Heavy alcohol Use						
Overall			67 (18.77)		
Alcohol alone			20 (5.60)		
Alcohol and virus			47 (13.17)		
Alcohol & HBV			25 (7.0)		
Alcohol & HCV			12 (3.36)		
Alcohol, HBV & HCV			11 (3.08)		
Negative for alcohol, smoking, HBV, HCV				65 (18.21)	

**Table 5:** Distribution of HCV genotypes in HCC patients and controls positive for HCV RNA.

	HCV related HCC (%)	HCV related chronic hepatitis (%)	*P*
Anti HCV positive/HCV RNA positive	68	55	
HCV Genotyping done	50 (73.53)	42 (73.36)	0.3637
Genotype 1	14 (28)	8 (19.05)	0.1994
Genotype 3	30 (60)	32 (76.19)	0.0633
Genotype 4	6 (12)	2 (4.76)	0.1379

**Table 6:** Distribution of HBV genotypes and viral loads in HCC patients.

	HCC, *n*=246 (%)
HBV DNA	
Negative	71 (38.9)
Positive	175 (71.1)
HBV genotype	
A	27 (15.4)
D	119 (68.0)
A+D	29 (16.6)
Log_10_ HBV DNA (copies/mL)	
Low (<5)	58 (33.1)
High (≥5)	117 (66.9)

Analysis of the risk factors for HBV markers showed that any marker positivity for HBV increases the risk of developing HCC by almost 27 times. HBsAg positivity or HBsAg negativity along with antibody positivity increases the risk of developing HCC by almost 18 folds or 17 folds, respectively. Any HCV marker positivity increases the risk of developing HCC by 10.6 times. The risk increases by 13.4 folds when cases with both anti-HCV and HCV RNA positivity were compared with the controls. Heavy alcohol use was found to double the risk of developing HCC when compared with the controls, whereas smoking was not found to be a risk factor (**Table 7**). Meanwhile, the risk increased by 55 times in alcoholic cases positive for any HBV serological marker compared with the controls. A 23-fold risk increase was observed when alcoholic cases with HBsAg positivity and antibody positivity were evaluated (**Table 8**).

**Table 7:** Distribution of HCC patients and controls according to major risk factors.

Risk factors		HCC cases *n*=357 (%)		Healthy controls *n*=120 (%)		OR (95% CI)	
HBV status							
Any Marker	246 (68.91)		9 (7.5)		27.33 (12.87-60.0)	
HBsAg+,antibody+	201 (56.30)		8 (6.67)		18.04 (8.22-41.19)	
HBsAg-,antibody+	45 (12.60)		1 (0.83)		17.16 (2.51-338.85)	
All markers-	111 (31.09)		111 (92.50)		Reference	
HCV Status							
Anti HCV + overall	76 (21.29)		3 (2.50)		10.55 (3.13-42.73)	
Anti HCV+,RNA+	66 (18.49)		2 (1.66)		13.38 (3.15-80.27)	
Anti HCV +,RNA-	10 (2.80)		1 (0.83)		3.43 (0.45-72.36)	
All markers -	281 (78.71)		117 (97.50)		Reference	
Heavy alcohol Use							
+	67 (18.77)		13 (10.83)		1.90 (0.97-3.78)	
-	290 (81.23)		107 (89.17)			
Smoking status							
Smokers (20/day for 10 years)	108 (30.25)		34 (28.33)		1.097 (0.6948- 1.732)	
Non Smokers	249 (69.75)		86 (61.67)			

**Table 8:** Profile of viral infection in HCC patients and controls with heavy alcohol use.

HBV and HCV status	HCC, *n*=67 (%)	Healthy controls, *n*=13 (%)	OR (95%CI)
HBV status			
Any marker+	55 (82.08)	1 (7.6)	55.00 (6.24-1245)
HbsAg+, antibody+	44 (65.67)	1 (7.6)	22.96 (2.77-502.29)
HbsAg-, antibody+	11 (16.42)	0	-
HCV status			
Overall	14 (20.90)	1 (7.6)	3.17 (0.37-70.71)
Anti HCV+, RNA +	12 (17.91)	1 (7.6)	2.62 (0.30-58.96)
Anti HCV+, RNA-	2 (2.98)	0	-

The HCC cases were distributed according to the presence and absence of cirrhosis and analyzed for the association of viral markers. Among the 253 HCC cases with cirrhosis, almost 70% were associated with HBV and/or HCV infection. Among the 104 HCC cases without cirrhosis, around 59% were associated with HBV and/or HCV infection. HBsAg positivity was observed in more than half of the cases in both cirrhotic and non-cirrhotic cases. Similarly, 21.7% of the cirrhotic HCC cases were positive for some HCV markers. An association between alcohol and virus was observed in 23.3% of the cirrhotic HCC cases. Moreover, cryptogenic HCC was documented in 14.6% of the cirrhotic cases and in almost 28.9% of the non-cirrhotic cases (**Table 9**). Positivity for any HBV marker increases the risk by almost 28 folds in the cirrhotic group and by 17.5 folds in the non-cirrhotic group. Positivity for any HCV marker increases the risk by 10.8 folds in the cirrhotic group. The risk remained in the cirrhotic cases of HCC when HCV markers were tested either alone or in combination. In cirrhotic cases of HCC, alcohol consumption increases the risk of developing HCC by 3 folds (**Table 10**).

**Table 9:** Prevalence of viral infection and heavy alcohol use among HCC patients with and without cirrhosis.

Etiological association	HCC with cirrhosis, n=253 (%)	HCC without cirrhosis, n=104 (%)	*P*
HBV marker			
Overall	176 (69.56)	61 (58.65)	0.02526
HbsAg+	154 (60.86)	54 (51.92)	0.06129
Only Anti HBe+	9 (3.56)	2 (1.92)	0.2266
Only IgG Anti HBc+	12 (4.74)	4 (3.85)	0.3716
HCV markers			
Overall	55 (21.74)	3 (2.88)	0.000000638
Anti HCV+, HCV RNA+	43 (17.00)	3 (2.88)	0.00004147
Anti HCV+, HCV RNA-	12 (4.74)	0	
HBV and HCV	24 (9.49)	0	
Overall	65 (25.69)	14 (13.46)	0.004946
Alcohol alone	8 (3.16)	9 (8.65)	0.01940
Alcohol and virus	59 (23.32)	6 (5.77)	0.00001529
Alcohol and HBV	31 (12.25)	4 (3.85)	0.005667
Alcohol and HBVand HCV	9 (3.56)	0	
Alcohol and HCV	16 (6.32)	1 (0.96)	0.01153
Negative for HBV, HCV and alcohol	37 (14.62)	30 (28.85)	0.001280

**Table 10:** Distribution of major risk factors in HCC patients with and without cirrhosis.

Risk factor	HCC with cirrhosis (*n*=253)				HCC without cirrhosis (*n*=104)		
	Cases positive	Healthy controls	OR (95%CI)		Cases positive	Healthy controls	OR(95%CI)
HBV status							
Any marker+	176 (69.56)	9 (7.5)	28.19 (13.03-62.96)		61(58.65)	9 (7.5)	17.50 (7.58-41.59)
HBsAg+, antibody+	154 (60.86)	8 (6.67)	21.78 (9.77-50.50)		54 (51.92)	8 (6.67)	15.12 (6.35-37.30)
HbsAg-, antibody+	21 (8.30)	1 (0.83)	10.77 (1.51-217.67)		6 (5.77)	1 (0.83)	7.29 (0.85-163.31)
HCV status							
Anti HCV+overall	55 (21.74)	3 (2.50)	10.83 (3.17-44.39)		3 (2.88)	3 (2.50)	1.16 (0.18-7.37)
Anti HCV+, HCV RNA+	43 (17.00)	2 (1.66)	12.08 (2.80-73.44)		3 (2.88)	2 (1.66)	1.75 (0.23-15.31)
Anti HCV+, HCV RNA-	12 (4.74)	1 (0.83)	5.93 (0.79-123.43)		0	1 (0.83)	-
Heavy alcohol Use		1 (0.83)				1 (0.83)	
+	65 (25.69)	13 (10.83)	2.85 (1.44-5.70)		14 (13.46)	13 (10.83)	1.28 (0.53-3.08)

### Biochemical profile of HCC and controls

Significant differences in biochemical parameters were observed between the two groups. The levels of white blood cells, differential leukocyte count (DLC)-P, DLC-L, DLC-M, platelets, international normalized level (INR), creatinine, aspartate aminotransferase (AST), alanine aminotransferase (ALT), alkaline phosphatase (ALP), and AFP were significantly increased in HCC. A twofold increase in risk was observed with the increased levels of creatinine, AST, ALP, and ALT in the HCC cases. An increased risk of 2.5 times was found in patients with AFP levels between 20 and 400 ng/mL, while a threefold increase in risk was observed in HCC patients with albumin levels less than 3.5 g/dl (**Tables 11**** and ****12**).

**Table 11:** Hematological and biochemical profile of HCC cases and controls.

Parameters	HCC (*n*=357)	Controls^†^ (*n*=357)	*P*
Hemoglobin (g/dL)	11.60± 2.10	11.30±2.80	0.1060
<10 mg/dL (%)	121 (33.89)	100 (28.0)	0.1050 [OR=1.32(0.95-1.83)]
WBC (×10^3^ /mm^3^)	6.60±0.40	6.50±0.20	0.0001*
DLC-P (%)	64.50±5.10	63.50±7.40	0.0359
DLC-L (%)	30.90±4.80	32.60±7.60	0.0004*
DLC-E (%)	3.05±1.40	2.90±5.10	0.5922
DLC-M (%)	1.50±0.80	1.90±1.80	0.0001*
Platelet (×10^4^/µL)	1.80±0.90	1.40±0.50	0.0001*
<1.5 lac/ µL (%)	115 (32.21)	105 (29.41)	0.4650 [OR=1.14 (0.82-1.59)]
INR	1.05±0.40	1.00±0.20	0.0350
Creatinine (mg/dL)	1.50±3.20	1.06±0.50	0.0105
>1.2 mg/dL (%)	101 (28.29)	69 (19.33)	0.0060* [OR=1.65 (1.14-2.37)]
AST (IU/L)	101.80±87.90	59.20±49.50	0.0001*
>2×ULN (%)	208 (58.26)	140 (39.21)	0.0000006*[OR=2.16 (1.59-2.95)]
ALT (IU/L)	67.10±46.30	57.30±47.40	0.0053
>2×ULN (%)	110 (30.81)	66 (18.48)	0.00018*[OR=1.96(1.36-2.83)]
ALP (IU/L)	4420.00±23.60	221.00±69.10	0.0001
>2×ULN (%)	172 (48.17)	135 (37.81)	0.0065*[OR=1.53(1.12-2.08)]
Bilirubin (mg/dL)	1.70±2.70	1.60±5.00	0.7396
>1.5 mg/dL (%)	192 (53.78)	103 (28.85)	0.00000*[OR=2.87(2.08-3.96)]
Albumin (g/dL)	3.60±0.70	3.70±0.90	0.0979
<3.5 g/dL (%)	265 (74.23)	174 (48.74)	0.00000*[OR=3.03(2.18-4.21)]
>3.5 g/dL (%)	92 (25.77)	183 (51.26)	0.00000*[OR=0.33(0.24-0.46)]
AFP (ng/mL)	6849.00±9652.00	5.90±1.20	0.0001*
<20 ng/mL (%)	68 (19.04)	311 (87.11)	0.00000*[OR=0.03(0.02-0.05)]
20-400 ng/mL (%)	96 (26.89)	46 (12.89)	0.0000043*[OR=2.49 (1.66-3.74)]
>400 ng/mL (%)	193 (54.06)	0 (0.0)	0.00000*[OR=UD]

†, chronic hepatitis cases without HCC;*,statistically significant

**Table 12:** Biochemical profile of HBV related HCC cases and controls.

Liver function indicators	HCC (*n*=246)	Controls^†^ (*n*=213)	*P*
ALT (U/liter)	70.20±49.80	51.80±33.20	<0.0001
AST (U/liter)	68.50±43.50	57.40±36.80	0.0036
ALP (U/liter)	407.00±522.00	237.00±60.50	<0.0001
ALB (g/liter)	3.10±0.60	3.70±1.08	<0.0001
T.BIL (mg/dL)	1.90±3.20	1.10±2.30	0.0026
Mean platelet count (10^4^/mm^3^)	1.32±0.40	1.89±0.50	<0.0001
INR (Sec)	1.03±0.50	1.02±0.17	0.7809
α-Fetoprotein (ng/mL)	4356.20±108.90	2.90±1.20	<0.0001

†, chronic hepatitis cases without HCC

### Radiological profile of HCC cases

Among the HCC cases, 55.7% had HCC in the right lobe, 30.8% in the left lobe, and 13.5% in both lobes of the liver. Three or more lesions were observed in 38.7%, two lesions were observed in 32.8%, and a single lesion in 28.6% of the HCC cases. More than half of the HCC patients (almost 58%) had a tumor size of ≥ 5 cm. Ultrasound images showed that approximately 38% of the cases were hypoechoic, 27.7% were heterogeneous, and 25.2% were hyperechoic. CT was possible in 314 of the total HCC cases, of which 48.7% were heterogeneous and 44% were hypodense. MRI was available for 81 cases of HCC. Portal vein invasion was observed in 43.1% of the cases (**Table 13**).

**Table 13:** Overall radiological profile of HCC cases.

Characteristics	HCC Cases (%)
Distribution of HCC	
Right lobe	199 (55.74)
Left lobe	110 (30.81)
Bilobar	48 (13.45)
Volume of HCC	
<50%	228 (63.86)
>50%	129 (36.13)
No. of Lesions	
1	102 (28.57)
2	117 (32.77)
≥3	138 (38.66)
Size of HCC, cm	
≤2	52 (14.57)
>-5	98 (27.45)
≥5	207 (57.98)
US appearance	
Heterogenous	99 (27.73)
Hypoechoic	137 (38.38)
Hyperechoic	90 (25.21)
Isoechoic	31 (8.68)
CT appearance^†^ (*n*=314)	
Hypodense	138 (43.95)
Heterogenous	153 (48.73)
Hyperdense	23 (7.32)
MRI appearance (*n*=81)	
T_2_ WI	
Hyperintense	62 (76.54)
Hypo/Isointense	19 (23.46)
T_1_ WI	
Hyperintense	28 (34.56)
Hypo/Isointense	53 (65.43)
Vascular Invasion	
Portal vein	154 (43.13)
PV with IVC/HC	19 (5.32)
IVC and or HV	6 (1.68)

IVC, inferior vena cava; HV, hepatic vein; PV, portal vein. †, Biphasic CT abdomen was done in 314 cases. MRI of the abdomen was done in 81 cases.

### Staging of HCC cases

HCC cases were staged according to the Okuda staging system, Cancer of the Liver Italian Program (CLIP) scoring system, and Barcelona Clinic Liver Cancer (BCLC) scoring system. Okuda stage 1 was observed in 37% of the cases, whereas stages 2 and 3 were found in 51.4% and 11.8% of the HCC cases, respectively. In the CLIP scoring system, 27.1% and 26.6% of the cases fall in CLIP scores 1 and 3, respectively. According to the BCLC scoring system, 44.3% of the cases were classified as stage A_1_, 22.4% as stage A_3_, 12% as stage A_4_, and 10.6% stage B. The percentage of death increased, and the mean survival decreased in ascending order of the stages in all the three staging systems (**Table 14**).

**Table 14:** Patients with different staging systems of HCC.

Staging	*n* (%)
Okuda stage	
I	132 (36.97)
II	184 (51.54)
III	42 (11.76)
CLIP score	
0	52 (14.56)
1	97 (27.17)
2	60 (16.81)
3	93 (26.65)
4-6	55 (15.41)
BCLC stage	
A_1_	158 (44.26)
A_2_	23 (6.44)
A_3_	80 (22.41)
A_4_	43 (12.04)
B	38 (10.64)
C	12 (3.36)
D	7 (1.97)

## Discussion

The male dominance observed in the present study is similar to that reported by many other studies from India and the rest of the world^[^^18^^-^^24^^]^. Similar to other studies, the maximum incidence of HCC occurred in patients in their 50s and 60s^[^^19^^,^^25^^,^^26^^]^.

Underlying cirrhosis was observed in almost 70.8% of the cases. The actual number may be slightly higher because of the non-invasive diagnostic modalities used in the present study to diagnose cirrhosis. Moreover, MRI/CT scans, which have higher sensitivity in detecting liver cirrhosis, were not performed in all cases. As suggested by previous studies^[^^27^^]^, HCC is accompanied by liver cirrhosis in 70% to 90% of the cases. However, many clinical studies have shown cirrhosis incidence of 30% to 80% in HCC^[^^18^^,^^28^^-^^31^^]^. The strong association between cirrhosis and HCC is supported by the evidence of its intermediating role in the pathogenesis of HCC because of chronic viral hepatitis^[^^32^^]^.

The clinical presentation of the HCC patients in this study was similar to that in previous studies. Both groups in the present study had a significant number of cases showing signs of ascites, hepatomegaly, pallor, pedal edema, icterus, and spleenomegaly. Symptoms such as abdominal pain, anorexia, weight loss, weakness, melena, and jaundice were present in more or less similar proportion in both categories. This finding is similar to that reported in an Indian study^[^^33^^]^. Furthermore, hepatic encephalopathy was observed in a very few cases of HCC. This result is similar to that reported by Wong et al.^[^^34^^]^, who found that Asian-American patients had a significantly lower frequency of hepatic encephalopathy compared with non-Asian Americans.

HBV association was found in 68.9% of the HCC cases, whereas HCV association was found in 21.3% of the HCC cases. This finding suggests that most HCC cases were the result of a hepatotropic virus-related chronic liver disease. This result is in accordance to the estimation that HBV is responsible for 50% to 80% of HCC cases worldwide, whereas 10% to 25% of the cases are thought to be caused by HCV infection^[^^35^^,^^36^^]^. Several studies reported that a dose-response relationship exists between the development of HCC and persistent HBV^[^^37^^,^^38^^]^.

The presence of alcoholic and cryptogenic HCC is more or less in accordance to the report of another Indian study^[^^39^^]^. A high section of HCC cases without known etiologic factors indicates the possibility of other unknown mechanisms for HCC development. Genotype 3 was the most common genotype, followed by genotypes 1 and 4 of HCV. This result is similar to that reported in other studies from India^[^^40^^-^^44^^]^. The results of the present study showed a significantly high OR for the development of HCC in HBsAg-positive patients and confirmed HBV as the main etiological agent associated to HCC in Indian scenario. An OR of 27.33 falls well in the range of findings of many case–control studies from other Asian countries, where the estimates of OR for HBsAg positivity range from 5 to 50^[^^45^^-^^48^^]^. A 13-fold risk increase was observed for patients with HCV RNA positivity and anti-HCV positivity, whereas an OR of approximately 3 was observed in individuals positive for anti-HCV but negative for HCV RNA. The reason is that the virus might have been cleared in these cases^[^^49^^]^. Other studies recorded that the OR range for HCV infection varies from non-significant to 8^[^^37^^,^^48^^,^^50^^]^. A significant correlation (OR=1.9) was observed between heavy alcohol consumption and the risk of developing HCC. This relationship is in agreement with previous studies^[^^42^^]^.

An increased OR was calculated in the HCC patients with reference to the controls in the heavy alcoholic group. However, considering that only one case in the control group was available, the findings may not depict the actual situation. Some epidemiological studies have described a high prevalence of HBV markers (27% to 81%) and HCV markers (50% to 77%) in alcoholic HCC patients compared with a background prevalence of approximately 5% and <1%, respectively. This finding suggests that a complex interaction exists between alcohol and viral infections in the etiology of HCC^[^^51^^,^^52^^]^. However, whether alcohol is a true carcinogen or if it acts as a cofactor in the presence of coexistent infection with HBV and/or HCV is still unclear.

The number of patients without cirrhosis (29.1%) in the present study is similar to those in Indian clinical and autopsy studies^[^^18^^,^^31^^,^^32^^,]^ and in a study of Kumar et al.^[^^4^^]^. An increased risk was observed for HBV marker irrespective of the cirrhosis status of the HCC patients. This result confirms that HBV is the major etiological factor associated with HCC development. These findings are in agreement with biological data. HBV plays a direct role in liver cell transformation; thus, it can lead to HCC without the development of cirrhosis^[^^12^^]^. Meanwhile, HCV markers alone or in combination were found to be a significant risk factor in cirrhotic HCC cases but not in non-cirrhotic HCC cases, which is similar to the scenario when heavy alcohol use was considered. The result may be that a large proportion of the non-cirrhotic HCC cases were of cryptogenic origin (28.9%) compared with the cirrhotic HCC group (14.6%). Hence, other risk factors, especially in the non-cirrhotic cases of HCC, should be investigated in future studies. Moreover, the carcinogenic effect of HCV in the absence of cirrhosis remains unknown^[^^4^^]^.

A strong positive correlation was noted between AST and ALT. This result is similar to the result reported by another Indian study on children^[^^53^^]^. Significance was observed for other parameters such as AST, INR, and creatinine levels, which were not previously reported as significant^[^^53^^]^. A total of 289 (80.96%) HCC cases showed raised AFP levels (>20 ng/ml). This result agrees with the study of Saini et al.^[^^54^^]^, where the percentage of HCC cases with raised AFP was 83%^[^^53^^]^.

Similar to the report of earlier Indian studies ^[^^4^^]^, a very high proportion of patients was found to have multiple lesions, larger tumor size, and advanced stage of the disease. Most of the lesions were hypoechoic (38.4%) or heterogeneous (48.7%), as previously reported. Approximately 58% of the cases had tumor size above 5 cm and a high incidence of vascular invasion with a very low resection rate. This result is similar to the findings of previous studies from India^[^^4^^,^^54^^]^.

## Conclusions

HBV and HCV are the major risk factors for HCC in India. All combination of HBV and HCV markers are risk factors for HCC. Although HCV RNA positivity and heavy alcohol use significantly increased the risk of developing HCC among cirrhotic patients, no significant risk increase was evident in the absence of cirrhosis. Alcohol alone is not a risk factor for HCC. Majority of the HCC cases have underlying cirrhosis. A raised biochemical profile was observed in HCC cases compared with the controls, although the clinical presentation was similar in both groups. At presentation, most of the patients have a large tumor (>5 cm) and multiple liver nodules. HCC is generally diagnosed at a later stage, making disease management difficult.
